# Use of rapid assessments of fishery bycatch of Humboldt penguins *Spheniscus humboldti* in Peru and Chile to help identify conservation priorities

**DOI:** 10.1098/rsos.250319

**Published:** 2025-08-06

**Authors:** Eduardo Segura-Cobeña, Joanna Alfaro-Shigueto, Valentina Colodro, Héctor Gutiérrez-Guzmán, Vania Arrese-Dávila, Ruben Torrejón-Zegarra, Lizzett Vega, Adrian Custodio-Uribe, Nelly Peña-Cutimbo, Joshimer Rodríguez-Salazar, David Messutto, Verónica Ugalde, Estaban Araya, Ian Tomás Andersen Muñoz, Eliana Alfaro-Cordova, Jeffrey C. Mangel

**Affiliations:** ^1^ Pro Delphinus, Lima District, Lima Region, Peru; ^2^ Carrera de Biología Marina, Universidad Científica del Sur, Lima, Peru; ^3^ Oikonos, Las Condes, Santiago, Chile; ^4^ Centre for Ecology and Conservation, University of Exeter, Penryn, UK

**Keywords:** Humboldt penguin, small-scale fishery, seabird bycatch, seabird–fishery interaction

## Abstract

The Humboldt penguin (*Spheniscus humboldti*) is a threatened species endemic to the Humboldt current system along the coasts of Peru and Chile. The species faces numerous and significant threats contributing to its declining population among which one of the most concerning is incidental catch (i.e. bycatch) by small-scale fisheries. This study assessed the bycatch of Humboldt penguins in small-scale surface and bottom gillnet and purse-seine fisheries using fisher surveys conducted across 39 landing sites (ports, coves and fishing villages) in Peru and Chile. A total of 779 fishers were surveyed. Results showed an estimated total of 4067 (±889 s.d.) penguins caught in 2023, with the highest bycatch associated with gillnets. Ports such as Tambo de Mora and San José in Peru and Coquimbo and San Antonio in Chile reported the most significant interactions. Spatial mapping demonstrated that areas with the most common bycatch events coincide with locations of larger penguin colonies. Multiple correspondence analysis revealed that larger mesh sizes (3–5 in (7.6 - 12.7 cm)) and certain target species, such as corvina drum (*Seriolella violacea*) and Peruvian grunt (*Anisotremus scapularis*), were strongly associated with reported bycatch events. This research underscores the urgent need for targeted conservation strategies, including bycatch mitigation measures, policy reforms and bi-national collaboration.

## 1. Introduction

The Humboldt penguin *Spheniscus humboldti* is a species endemic to the nutrient-rich waters of the Humboldt current system that flows northwards along the west coast of South America [1]. The species is categorized as vulnerable (VU) by the International Union for Conservation of Nature (IUCN) [2] and is included in appendix I of the Convention on International Trade in Endangered Species of Wild Fauna and Flora (CITES). It is also classified as endangered by the government of Peru (D.S. N°004-2014-MINAGRI) and as vulnerable by Chile (Decreto N°1, 25 July 2024 Ministerio del Medio Ambiente). Furthermore, population estimates projected that the species would decline at 7% per year [3] and is expected to go extinct in *ca* 59 years (between 35 and 85 years) [4], suggesting that Humboldt penguins should be considered as endangered following the IUCN Red List classification criteria.

The species is distributed along the coasts of Peru and Chile, from La Foca Island (05°12′ S, 81°12′ W) to Metalqui Island (42°12′ S, 74°09′ W) [5,6]. Eighty colonies of Humboldt penguins have been identified: 42 in Peru and 38 in Chile [7]. Penguins use colonies for breeding and during moult seasons [3]. Humboldt penguins typically forage close to the colony (travel distances can vary from less than 100 m to 15−40 km), and for this reason, they are considered sedentary [3,8,9].

Vianna *et al.* [7] estimated a total population size of 36 982 Humboldt penguins between Peru and Chile based upon reviews of other studies from 1997 to 2011, but with a substantial decrease in the population since then. Threats affecting the species include habitat loss, invasive species, contamination at the colonies, climate change, avian influenza and fishery interactions [10,11]. To improve resilience of penguin populations to climate change and El Niño Southern Oscillation (ENSO) events, other threats need to be substantially reduced. Incidental mortality in fisheries has been identified as one threat that could be addressed in the near-term [12].

Considering that in Peru and Chile there are approximately 23 138 and 12 669 small-scale fishing vessels, respectively [13,14], the impact of interactions with these large fleets may be significant for Humboldt penguins. Studies in Chile have shown that the most significant interactions happen with purse-seine and gillnet fisheries [15–19]. In Peru, evidence of incidental catch and mortality of penguins in gillnet fisheries has been noted since the early 1980s [4,5,20,21]. In Chile, penguin bycatch has been registered in several types of fishing gear such as purse-seines, longlines and gillnets; however it occurs more frequently with gillnets [12,15]. There is, however, relatively limited published literature on the Humboldt penguin and its vulnerability to bycatch. This is possibly related to Humboldt penguin bycatch interactions with small-scale fisheries which are highly dispersed along a vast coast, making them difficult to monitor (e.g. lack of mandatory independent onboard observer programs), characterize, quantify and track their effects on penguin populations [5,16,17]. McGill *et al.* [3] conducted a population viability analysis of the Humboldt penguin population which highlights the limited information about bycatch of the species, especially the possibility of overlap with breeding areas.

The use of fisher surveys to develop bycatch assessments, also called rapid bycatch assessments, have been implemented for various marine fauna [22–25]. Rapid assessments are an affordable and relatively fast methodology compared to the implementation of onboard monitoring programmes [26]. This methodology has been used to identify important areas for conservation and mitigation, e.g. the identification of areas that needed of further knowledge about leatherbacks in the Eastern Pacific [24]; to identify ports in Peru that required information for turtle bycatch and highlighting gillnets and longlines as important fishing gears related to bycatch [16,27]; and to identify priority fishing ports to use LED lights as a sea turtle bycatch mitigation technology [28].

The objective of this study was to consolidate information obtained through fisher surveys to assess Humboldt penguin bycatch (i.e. rapid bycatch assessment) and to identify areas of highest bycatch occurrence along the Peru and Chile coastlines. Areas identified as having high interaction rates could serve as hotspots for further conservation measures (i.e. where to test or implement bycatch mitigation measures), including more in-depth research to fill information gaps and refine estimates of bycatch mortality. The information from this study, collected throughout the distribution of the species and based on the local knowledge of fishers, can serve to estimate penguin bycatch and mortality and as an input to develop and promote the use of best practice guidelines for surface and bottom gillnet and purse-seine fisheries.

## 2. Methodolody

### 2.1. Study area

We collected information from local fishers and conducted semi-structured surveys to surface gillnet, bottom gillnet and purse-seine fishers in Peru and Chile. Surveys in Peru were completed between April and June 2023, and those in Chile were completed between August and October 2023. A total of 17 fishing villages, coves or landing sites (for brevity hereafter all referred to as ‘ports’) were assessed in Peru and 22 ports in Chile (table 1). Ports were selected based upon their occurrence within the distribution of the species (including their proximity to known penguin colonies) and the presence of sizeable small-scale gillnet or purse seine fleets.

**Table 1.** Summary of the number of surveys carried per port per gear and of Humboldt penguin bycatch per port by fishing gear. (Scaled bycatch was evaluated using the mean bycatch per boat per port and the half of the fleet size of port; % fishers reporting bycatch is the per cent of fishers from total surveys at the port that said that they had penguin bycatch events.)

**Country**	Port	Total interviews	Surface gillnet					Bottom gillnet					Purse-seine				
			Fleet size	No. interviews	% Fishers reporting bycatch	Bycatch per vessel per year (mean ± s.d.)	Scaled bycatch (mean ± s.d.)	Fleet size	No. interviews	Fishers reporting bycatch	Bycatch per vessel per year (mean ± s.d.)	Scaled bycatch (mean ± s.d.)	Fleet size	No. interviews	Fishers reporting bycatch	Bycatch per vessel per year (mean ± s.d.)	Scaled bycatch (mean ± s.d.)
Peru	Sechura	51	15	0	—	—	—	20	21	2	0.1 ± 0.3	2 ± 1	400	30	2	0.9 ± 3.2	355 ± 65
	San Jose	37	80	5	3	0.6 ± 0.5	47 ± 4	300	29	11	1.8 ± 4.6	528 ± 80	202	3	0	0 ± 0	0 ± 0
	Santa Rosa	17	150	2	0	0 ± 0	0 ± 0	—	0	—	—	—	220	15	2	0.7 ± 2.1	163 ± 31
	Pacasmayo	30	60	10	8	1.6 ± 1.5	98 ± 12	60	19	15	3.7 ± 5.2	224 ± 40	—	1	0	0 ± 0	0 ± 0
	Salaverry	30	61	18	9	0.8 ± 0.9	50 ± 7	5	12	7	2.1 ± 3.7	10 ± 8	—	0	—	—	—
	Chimbote	39	50	11	5	1 ± 1.3	52 ± 9	7	16	8	1.5 ± 2.1	10 ± 6	20	12	0	0 ± 0	0 ± 0
	Huarmey	25	35	6	3	0.5 ± 0.5	17 ± 3	35	19	12	1.5 ± 2.4	51 ± 14	73	0	—	—	—
	Huacho	21	45	21	11	0.8 ± 1.6	38 ± 10	45	0	—	—	—	20	0	—	—	—
	Chancay	30	64	17	11	2.5 ± 4.6	159 ± 37	64	13	7	1.4 ± 2.8	90 ± 22	—	0	—	—	—
	Ancón	31	247	26	13	0.7 ± 1	181 ± 15	5	5	2	0.4 ± 0.5	2 ± 1	—	0	—	—	—
	Pucusana	39	60	26	12	1.2 ± 2.9	69 ± 22	20	3	2	2 ± 1.5	41 ± 7	10	10	3	0.5 ± 1	5 ± 3
	Tambo de Mora	16	13	7	6	12.5 ± 15.9	162 ± 57	13	9	3	1.2 ± 2	16 ± 7	—	0	—	—	—
	San Andrés	62	30	21	5	0.2 ± 0.5	6 ± 3	15	10	7	1.6 ± 1.8	24 ± 7	129	31	5	0.3 ± 0.8	41 ± 9
	San Juan de Marcona	13	12	1	0	0 ± 0	0 ± 0	12	12	6	1.1 ± 1.5	13 ± 5	10	0	—	—	—
	Puerto de Lomas	27	25	19	14	3.9 ± 5.5	97 ± 27	25	8	4	1 ± 1.3	25 ± 6	—	0	—	—	—
	Atico	11	6	0	—	—	—	—	0	—	—	—	29	11	3	0.6 ± 1.1	16 ± 6
	Ilo	33	—	0	—	—	—	—	1	0	0 ± 0	0 ± 0	110	32	5	0.3 ± 0.7	31 ± 7
	Total						976 ± 208					1036 ± 205					612 ± 121
Chile	Arica	9	55	4	2	0.8 ± 0.8	42 ± 6	120	5	4	1.3 ± 1	154 ± 11	—	0	—	—	—
	Riquelme	12	13	9	3	0.4 ± 0.8	6 ± 3	13	2	1	0.5 ± 0.4	7 ± 1	—	1	1	0 ± 0	0 ± 0
	Cavancha	6	6	2	0	0 ± 0	0 ± 0	8	4	3	1 ± 0.9	8 ± 3	—	0	—	—	—
	San Pedro	6	—	0	—	—	—	7	6	2	0.2 ± 0.3	1 ± 1	—	0	—	—	—
	Hornitos	4	1	1	0	0 ± 0	0 ± 0	3	3	1	0.7 ± 0.8	2 ± 1	—	0	—	—	—
	Mejillones	6	20	3	2	1 ± 0.3	21 ± 2	2	2	2	2.5 ± 0.3	5 ± 0	—	1	1	0 ± 0	0 ± 0
	Lagarto	3	2	2	1	1 ± 0.7	2 ± 1	—	1	0	0 ± 0	0 ± 0	—	0	—	—	—
	Antofagasta	12	59	5	5	2 ± 1.7	121 ± 13	—	1	0	0 ± 0	0 ± 0	3	6	4	1 ± 1.1	3 ± 2
	Chañaral de Aceituno	4	5	2	0	0 ± 0	0 ± 0	5	2	0	0 ± 0	0 ± 0	—	0	—	—	—
	Punta de Choros	9	8	2	0	0 ± 0	0 ± 0	11	7	1	0.1 ± 0.3	2 ± 1	2	0	—	—	—
	Chungungo	6	7	3	0	0 ± 0	0 ± 0	12	3	0	0 ± 0	0 ± 0	—	0	—	—	—
	Coquimbo	21	79	7	3	7.3 ± 13.8	579 ± 123	79	5	2	0.4 ± 0.6	32 ± 6	30	9	6	7.5 ± 9.7	224 ± 53
	Totoralillo Norte	4	4	1	0	0 ± 0	0 ± 0	6	3	0	0 ± 0	0 ± 0	—	0	—	—	—
	Guanaqueros	14	—	0	—	—	—	—	0	—	—	—	17	14	1	0 ± 0	0 ± 0
	Tongoy	12	21	6	1	4.2 ± 7.6	89 ± 35	—	2	0	0 ± 0	0 ± 0	—	4	0	0 ± 0	0 ± 0
	San Pedro Los Vilos	13	—	2	0	0 ± 0	0 ± 0	7	11	3	5.1 ± 11.7	36 ± 31	—	0	—	—	—
	Pichidangui	13	6	9	1	0.2 ± 0.5	1 ± 1	6	4	2	0.5 ± 0.5	3 ± 1	—	0	—	—	—
	Papudo	9	—	0	—	—	—	12	9	1	0.1 ± 0.3	1 ± 1	—	0	—	—	—
	Higuerillas	15	—	0	—	—	—	18	15	5	0.5 ± 1.3	8 ± 5	—	0	—	—	—
	El Membrillo	8	—	0	—	—	—	18	8	0	0 ± 0	0 ± 0	—	0	—	—	—
	Diego Portales	36	—	0	—	—	—	52	35	1	0 ± 0	0 ± 0	—	1	0	0 ± 0	0 ± 0
	San Antonio	45	8	28	14	12.3 ± 19.2	98 ± 54	—	15	4	0.9 ± 2.2	0 ± 0	—	2	0	0 ± 0	0 ± 0
	Total						958 ± 238					258 ± 62					227 ± 55

### 2.2. Survey design and data collection

Surveys consisted of 24 questions (open and closed) focussed on collecting information on fishing effort and penguin bycatch. Surveys were conducted with randomly selected vessel captains or crew who were willing to participate, and with only one representative survey per vessel to reduce possible duplication. Surveys were divided into four sections: fisher background (Q1–Q6); vessel characteristics (Q7–Q9); fishing gear and capture (Q10–Q15); and the last part of the survey (Q16–Q23) included questions related to penguin bycatch in the fishing gear most used by the fisher (the surveys’ questions are detailed in the electronic supplementary material, S1). Illustrated identification guides assisted fishers in penguin species identification. During the surveys, we showed respondents a map of their fishing area and asked them to draw a polygon indicating areas where penguin bycatch happened to them. An additional section was completed by the interviewer about the perceived honesty/reliability of the respondent. An honest interviewee was considered as one who does not hesitate when answering, who does not change their opinion, does not evade questions and whose responses were consistent. Responses from fishers considered by the interviewer to be unreliable were eliminated from the analysis.

Prior to each survey, participants verbally consented to participate and were informed that their participation was voluntary and anonymous, that the information would not be shared with anyone and was for research purposes only. Survey questions were designed and tested to ensure that they were not overly intrusive. All researchers involved in surveys were fluent in the language and colloquial terminology of the study area. Research ethics were approved by the Universidad Cientifica del Sur Ethics Committee (051−2023-PRO99).

### 2.3. Data analysis

#### 2.3.1. Penguin interactions

Reports of penguin bycatch were only included in the analysis when fishers answered the penguin bycatch question (Q16). For condition and fate questions (Q22 and Q23), fishers could give more than one answer, but for analysis, we only include the one that they considered the most common condition or fate. For all questions, a ‘no response’ (i.e. the fisher did not answer the question) was counted and included in the analysis as ‘unknown’.

Using the QGIS software [29], the polygons drawn by fisher where bycatch is more common to happen were digitized and georeferenced and combined into a map. Then, to identify areas where penguin bycatch was more common, we calculated the polygon density (number of polygons per raster pixel) along the coast.

#### 2.3.2. Penguin bycatch

Penguin bycatch estimates were calculated based upon fisher responses (Q16–Q23). We assigned zero penguins caught when fishers answered ‘no’ to penguin bycatch question (Q16) or ‘yes’ but without reporting the number (Q18). When fishers report a range of penguins caught (Q18), we used the minimum value of the range provided in analyses. We consider that fisher’s bycatch responses were related to the port where the survey was conducted. However, we acknowledge that fishers sometimes operate from multiple ports and report bycatch related to different locations.

To estimate penguin bycatch during 2023, we used bootstrap analyses, available in R package ‘boot’, with 1000 replications [30]. The resampling unit was the number of penguins incidentally caught per vessel as reported by fishers for the year 2023. We also incorporated within the analysis observations with zero captures which corresponded to those fishers who answered 'no' to having incidental penguin bycatch. Bycatch estimates were calculated as mean penguin bycatch per year per vessel for each type of fishing gear (gillnet or purse-seine) for each port. To obtain an estimate of the total penguin bycatch per port, we extrapolated the mean penguin bycatch obtained from the bootstrap to the port fleet size per type of fishing gear. The associated s.d. was obtained by multiplying the bootstrap standard deviation by the square root of the fleet size. To avoid possible overestimation of bycatch owing to inaccurate information on the size of the fishing fleets, at each port, we determined the number of active vessels (not just registered vessels, which are usually higher in number) using surface gillnet, bottom gillnet or purse-seine. For those ports where this information was unavailable, the total active surface gillnet, bottom gillnet and purse-seine fleet sizes were obtained from the ENEPA III (2018) (available at: https://hdl.handle.net/20.500.12958/3300) surveys of small-scale fisheries for Peru and from Registro Pesquero Artesanal for Chile (available at: https://registropublico.sernapesca.cl/reportes/regembarcaciones_publico/index.php). As the gillnet fleet size census in Chile does not detail if fishers use surface or bottom gillnet, we considered each of them as 50% of the gillnet fleet size.

#### 2.3.3. Multiple correspondence analysis

We applied the multiple correspondence analysis (MCA) to visualize associations between penguin bycatch (yes or no) with target species and mesh size, with the objective of generating graphical representations that facilitate data interpretation. All the variables were considered active variables, used to construct the main dimensions of the analysis and explain variation among observations. The active variables represent the primary factors structuring the relationships between observations. MCA was performed using the ‘FactoMineR’ package [31] of the R software, version 4.1.2 [32].

## 3. Results

### 3.1. Summary of fishing results

A total of 779 surveys were conducted in 39 ports across Peru and Chile (table 1). In Peru, we conducted 512 surveys, 190 with surface gillnets users and 177 with bottom gillnet users conducted in 16 ports assessed. Additionally, 145 surveys were with purse-seine fishers from nine ports. In Chile, we conducted 267 surveys, 86 with surface gillnet users and 143 with bottom gillnet users in 17 ports. Additionally, 38 surveys were with purse-seine fishers from eight ports. Between Peru and Chile, 25 surveys were conducted with users of mid-water gillnets, but given this small sample size, these data were not considered for further analysis.

### 3.2. Penguin bycatch

Penguin bycatch was reported in all ports from Peru surveyed. On the other hand, 6 of 23 ports (26%) from Chile did not report penguin bycatch (Chañaral de Aceituno, Chungungo, Totoralillo Norte, Guanaqueros, El membrillo, Diego Portales). From the 512 fishers interviewed in Peru, 206 (40.2%) reported Humboldt penguin bycatch, and from the 267 fishers interviewed in Chile, 77 (28.8%) reported Humboldt penguin bycatch (table 1). Combining information from both countries and fishing gears we estimated that approximately 4067 (±889 s.d.) penguins were captured in 2023 (table 1) of which 2624 (±534 s.d.) were captured in Peru and 1443 (±355 s.d.) were captured in Chile.

The highest reported penguin bycatch was by users of both kind of gillnets (surface and bottom) in Peru, but only of surface gillnets in Chile. In Peru, Tambo de Mora had the highest average reported bycatch with 12.5 penguins per vessel per year with surface gillnets (table 1), while the other ports had an average of 1–3 penguins per vessel per year. In total, 976 penguins were estimated bycaught in Peru in 2023 by fishers using surface gillnets. In Chile, San Antonio had the highest average reported bycatch with 12.3 penguins per vessel per year, and estimated a total of 958 penguins bycaught in the country in 2023 with surface gillnets.

Bottom gillnet users had a similar reported penguin bycatch per year, compared to surface gillnets in Peru. The port of Pacasmayo had the highest average reported bycatch per vessel with 3.7 penguins caught per year, while the port of Salaverry was the second highest with 2.1 penguins reported bycaught per vessel per year. However, in three ports from Peru (Santa Rosa, Huacho and Atico) no bottom gillnet users were surveyed, and no users of bottom gillnets from Ilo responded that penguins were bycaught. In total, 1036 penguins were reported bycaught in Peru during 2023 using bottom gillnets. In Chile, a lower level of bycatch was reported compared to surface gillnets. Fishers from eight ports responded that no penguins were bycaught using bottom gillnets. In the port of Guanaqueros, no bottom gillnet fishers were surveyed. In Chile, the highest average reported bycatch was in San Pedro Los Vilos with 5.1 penguins per vessel, followed by Mejillones with 2.5 penguins per vessel. In total, 258 penguins were reported bycaught in Chile in the last year using bottom gillnets.

Reported penguin bycatch in purse-seines was lower in Peru than in Chile. In Peru, six of the nine ports surveyed had fishers who reported penguin bycatch with purse-seines, while in Chile, fishers from two of the five ports assessed which use that gear reported penguin bycatch. In Peru, the average penguin bycatch reported by fishers using purse-seines was less than one penguin per vessel per year, with Sechura and Santa Rosa being the ports with the highest values, with 0.89 and 0.74 penguins caught per vessel per year, respectively. In Chile, Coquimbo and Antofagasta were the only ports with reports of penguin bycatch, from the five ports evaluated, with an average of 7.46 and 0.99 penguins per vessel per year, respectively. A total of 612 and 227 penguins were estimated bycaught with purse-seines in Peru and Chile, respectively.

#### 3.2.1. Spatial-temporal patterns of penguin bycatch

The periods of highest bycatch as indicated by fishers varied among ports (table 2). In ports from northern Peru and central Chile, fishers reported low probabilities of bycatch throughout the year. On the other hand, ports located in central and southern Peru and northern Chile reported higher rates of bycatch events during the austral summer (December–March).

**Table 2.** Percentage of the most mentioned months with Humboldt penguin bycatch events per port. Data are the percentage of fishers that mentioned the months when events occur from the total number of surveys per port.

Country	Ports	Jan	Feb	Mar	Apr	May	Jun	Jul	Aug	Sep	Oct	Nov	Dec
Peru	Sechura	2	2	2	0	0	2	2	0	0	0	2	0
	San Jose	5	5	0	8	0	3	5	0	8	3	5	5
	Santa Rosa	6	6	6	6	0	0	0	6	0	0	0	0
	Pacasmayo	47	50	40	20	10	7	7	3	3	3	3	7
	Salaverry	33	33	20	13	7	13	17	17	13	13	10	30
	Chimbote	0	0	0	5	3	3	3	3	3	5	5	8
	Huarmey	16	16	24	12	0	0	8	4	0	0	4	4
	Huacho	24	29	10	10	10	5	5	5	0	0	0	0
	Chancay	13	20	10	13	0	3	7	3	0	3	0	3
	Ancon	19	19	13	6	3	13	13	13	3	3	3	6
	Pucusana	5	5	0	3	5	5	0	3	8	3	0	3
	Tambo de Mora	25	19	6	13	31	13	13	6	6	6	13	6
	San Andrés	6	8	13	11	5	3	2	2	2	2	3	5
	San Juan de Marcona	31	15	0	0	0	0	0	0	0	0	0	0
	Puerto de Lomas	30	41	41	19	11	15	19	15	4	4	0	4
	Atico	18	18	9	0	0	9	9	0	0	0	0	0
	Ilo	3	3	3	3	3	6	9	12	12	12	6	3
Chile	Arica	0	11	0	11	11	22	11	11	0	11	11	0
	Riquelme	0	8	8	0	8	0	0	0	0	0	8	0
	Cavancha	33	33	50	17	17	17	17	17	17	17	17	17
	San Pedro	17	17	17	17	17	17	17	17	17	17	17	17
	Hornitos	0	0	0	0	0	0	25	0	0	0	0	0
	Mejillones	50	50	17	17	0	0	0	17	50	0	17	17
	Lagarto	0	0	0	0	0	0	0	0	33	0	0	33
	Antofagasta	8	8	0	0	0	0	0	8	17	25	25	17
	Chañaral de Aceituno	0	0	0	0	0	0	0	0	0	0	0	0
	Punta de Choros	11	0	0	0	0	0	0	0	0	0	0	11
	Chungungo	0	0	0	0	0	0	0	0	0	0	0	0
	Coquimbo	48	48	43	5	5	5	5	5	5	10	10	10
	Totoralillo Norte	0	0	0	0	0	0	0	0	0	0	0	0
	Guanaqueros	7	7	7	0	0	0	0	0	0	0	0	0
	Tongoy	8	8	8	8	0	0	0	0	0	0	0	0
	San Pedro Los Vilos	23	23	15	8	0	0	0	0	0	0	0	0
	Pichidangui	15	15	15	0	0	0	0	0	0	0	0	0
	Papudo	11	11	0	0	0	0	0	0	0	0	0	0
	Higuerillas	7	7	0	0	0	13	13	0	0	0	0	0
	El Membrillo	0	0	0	0	0	0	0	0	0	0	0	0
	Diego Portales	0	0	0	0	0	0	0	0	0	0	0	0
	San Antonio	36	38	33	29	24	20	11	0	0	0	0	0

**Figure 1.**
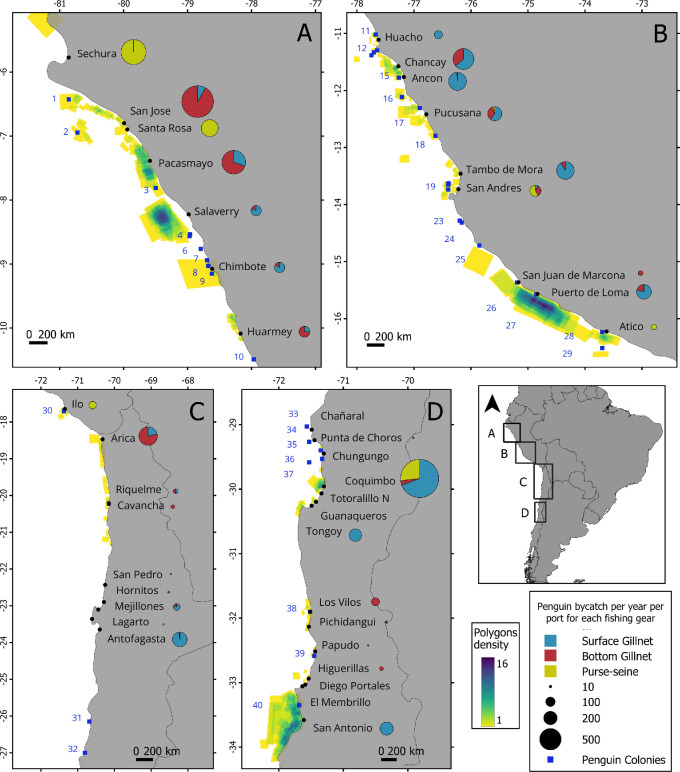
Total Humboldt penguin bycatch scaled by the fleet size of each port (circles: blue for surface gillnet, red for bottom gillnet and yellow for purse-seine) and polygon density (number of polygons per rater pixel) indicate spatial distribution of areas where Humboldt penguin bycatch is most common. A, North-central Peru; B, South-central Peru; C, northen Chile; and D, central Chile. Black points show port where surveys were conducted, blue squares show penguin colonies (1 Lobos de Tierra Island; 2 Lobos de Afuera Island; 3 Isla Macabi; 4 North and South Guañape Island; 6 Chao Island; 7 Corcovado Island; 8 Santa Island; 9 North Ferrol Island; 10 Punta Colorado; 11 Don Martín Island; 12 include Punta Salinas, Huampanu Island and Mazorca Island; 15 Pescadores Island; 16 Cavinzas Island; 17 Pachacamac Island; 18 Asia Island; 19 includes North, Center and South Chincha Islands and Ballesta Island; 23 La Vieja Island; 24 Santa Rosa Island; 25 Punta Lomitas; 26 Punta San Juan; 27 Punta Lomas; 28 Punta Atico; 29 Punta La Chira; 30 Punta Coles; 31 Pan de Azucar Island; 32 Ramadas Island; 33 Chañaral Island; 34 Choros Island; 35 Chugungo Island; 36 Tilgo Island; 37 Pajaros 1 Island; 38 Huevos Island; 39 Cachagua Island; 40 Pajara Niño Island).

In terms of spatial patterns of fishery interactions, fisher responses indicated areas with higher probabilities of bycatch events. In both Peru and Chile, bycatch events were more frequently reported to occur around guano islands and capes with penguin colonies. In Peru, the principal areas where penguin bycatch was reported were north of Macabi and Guañape islands (figure 1A), between Huampanu and Pescadores islands, and south of Punta San Juan (figure 1B). In Chile, the principal areas were in the central area in front of Coquimbo near to Pajaros 1 island and in the waters around San Antonio and south of Pajaro Niño island (figure 1C,D).

#### 3.2.2. Penguin condition and fate after bycatch events

A high percentage of the respondents chose not to answer questions related to the condition (non-response rate per port = 47–86%) and fate (non-response rate per port = 50–86%) of penguins caught as bycatch (tables 3 and 4). However, penguins found dead in both surface and bottom gillnets were more frequently reported in Peru than in Chile. For those fishers who did respond to these questions, penguins were most commonly reported to be retrieved dead (response rate per port = 7–41%) and the most common fates were released alive (response rate per port = 6.2–26.3%) or discarded dead (response rate per port = 7.6–23.2%). Fishers from Peru also mentioned that bycaught penguins could be eaten, sold, or used as pets, but fishers from Chile did not provide answers other than released alive or discarded dead.

**Table 3.** Condition of penguins during bycatch events. (Numbers are the percentage of fishers from total surveys per country.)

Fishery	Country	Number of surveys	Condition				No response
			Good condition	Injured	Poor condition	Dead	
Surface gillnet	Peru	190	26.3	2.6	3.2	35.3	47.4
	Chile	86	9.3	3.5	0.0	19.8	67.4
Bottom gillnet	Peru	177	9.6	0.6	2.8	41.8	51.4
	Chile	143	5.6	0.7	0.0	16.1	77.6
Purse-seine	Peru	145	4.8	0.7	0.0	10.3	86.2
	Chile	38	18.4	5.3	0.0	7.9	68.4

**Table 4.** Fate of penguins after bycatch events. (Numbers are the percentage of fishers from total surveys per country.)

Fishery	Country	Number of surveys	Fate					No response
			Released alive	Discarded dead	Eaten	Sold	As pet	
Surface gillnet	Peru	190	24.7	23.2	16.8	0.5	1.1	50.5
	Chile	86	19.8	16.3	0.0	0.0	0.0	64.0
Bottom gillnet	Peru	177	9.6	27.7	13.6	1.1	1.1	57.1
	Chile	143	7.7	14.0	0.0	0.0	0.0	78.3
Purse-seine	Peru	145	6.2	7.6	3.4	0.0	0.0	86.2
	Chile	38	26.3	7.9	0.0	0.0	0.0	65.8

#### 3.2.3. Target species and net mesh size

**Figure 2.**
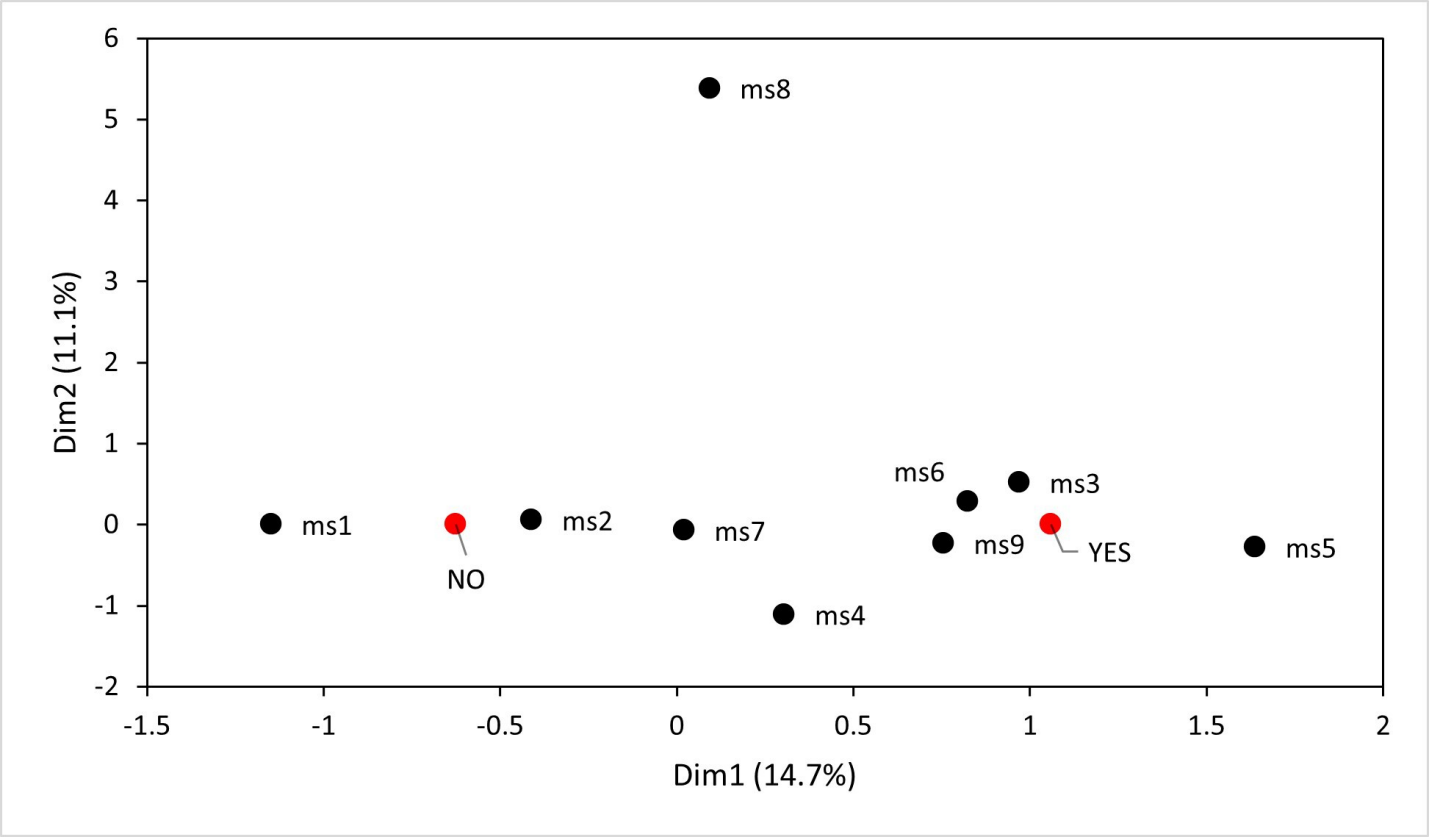
MCA showing the distribution of the variables mesh size and penguin bycatch events along dimension 1 (Dim1, explaining 14,7% of the variance) and dimension 2 (Dim2, explaining 11.1% of the variance). Where the variables related to fishing are in black points: mesh size (ms) = ms1–ms9, corresponding to 1–9 in (2.54–22.86 cm), with ms9 indicating 9 in (22.86 cm) or larger. Variables related to interaction of artisanal fishery with Humboldt penguin are in red points: YES, meaning fishers had at least 1 Humbolt penguin bycatch; and NO, meaning fishers did not had Humboldt penguin bycatch.

**Figure 3.**
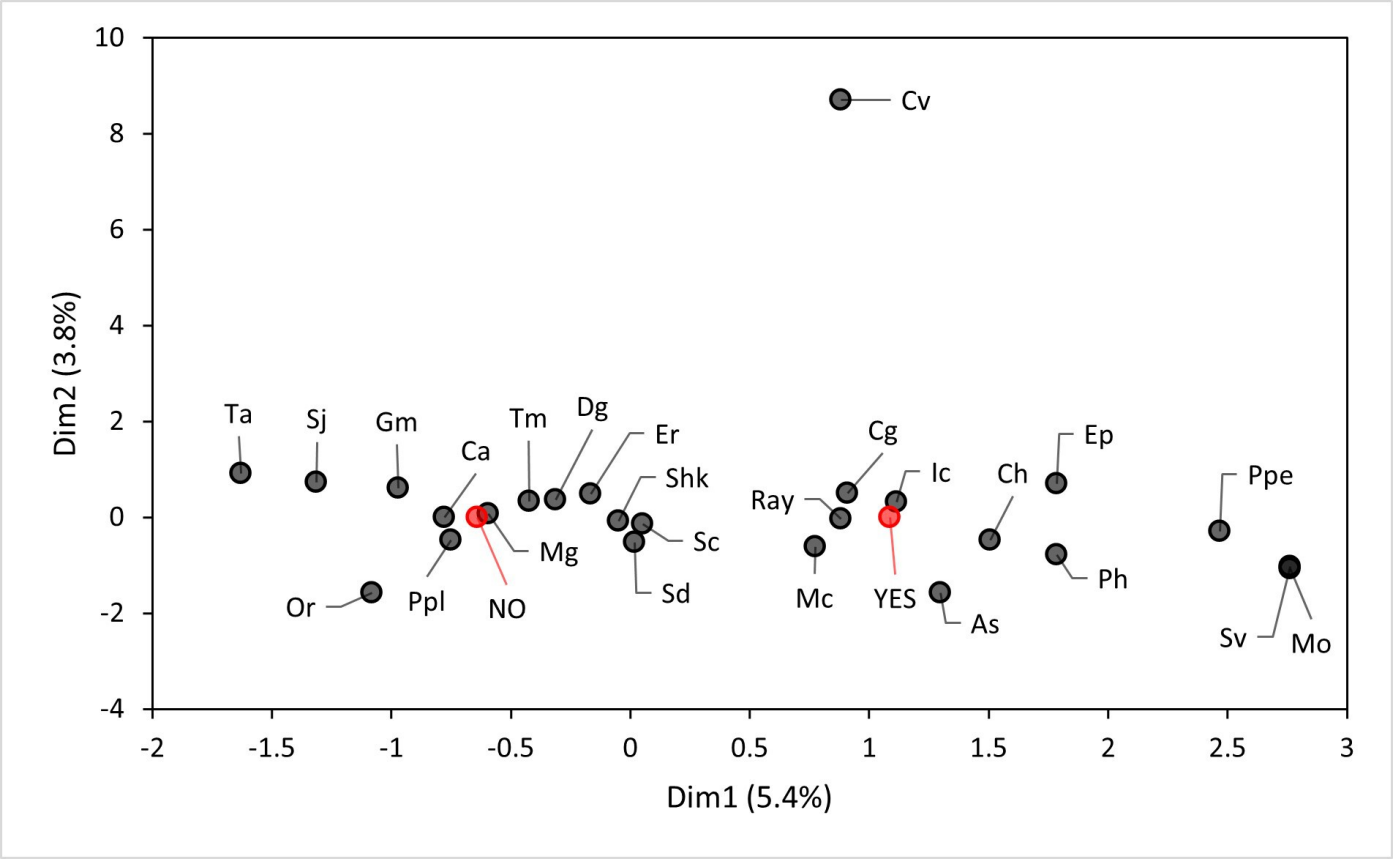
MCA showing the distribution of the variables target species and penguin bycatch events along dimension 1 (Dim1, explaining 5.4% of the variance) and dimension 2 (Dim2, explaining 3.8% of the variance). Where the variables related to fishing are target species, with black points: target species = Ta (*Thunnus alalunga*), Gm (*Genypterus maculatus*), Ic (*Isacia conceptionis*), Or (*Odontesthes regia regia*), Sj (*Scomber japonicus*), Ch (*Coryphaena hippurus*), Cg (*Cilus gilberti*), Sc (*Sarda chilensis*), Mc (*Mugil cephalus*), Tm (*Trachurus murphyo*), Dg (*Dosidicus gigas*), Sd (*Sciaena deliciosa*), Ppl (*Pseudobatos planiceps*), Sv (*Seriolella violacea*), Er (*Engraulis ringens*), Ppe (*Paralonchurus peruanus*), Ep (*Etropus peruvianus*), As (*Anisotremus scapularis*), Cv (*Chirodactylus variegatus*), Ca (*Cynoscion analis*), Ray (Various rays spp.), Shk (Various sharks spp.), Mo (*Menticirrhus ophicephalus*), Mg (*Merluccius gayi*) and Ph (*Paralabrax humeralis*) . Variables related to the interaction of artisanal fishery with Humboldt penguins, with red points: YES, meaning fishers had at least one Humboldt penguin bycatch; and NO, meaning fishers did not had Humboldt penguin bycatch.

Based on the MCA, two main associations were observed. The analysis related to mesh size and penguin bycatch events (‘yes’ or ‘no’) showed that certain mesh sizes (e.g. 3′, 5′ and 6′ (7.6 cm, 12.7 cm, 15.2 cm)) tended to group at the direction of the ‘yes’ category, with 14.7% of the variability explained (Dim 1, figure 2). This suggests a potential association between these mesh sizes and the occurrence of penguin bycatch, while other mesh sizes (e.g. 1′ and 2′ (2.5 cm, 5.0 cm)) aligned more with the ‘no’ category, indicating a lower likelihood of bycatch (figure 2). For the analysis focused on target species and bycatch presence, specific target species, such as corvina drum *Seriolella violacea*, rays and Peruvian grunt *Anisotremus scapularis*, were associated with the ‘yes’ category, but with low percentages (5.4%) of the variability explained (Dim 1, figure 3). This potentially indicates a higher bycatch risk for penguins when targeting these species. By contrast, other species like Chilean silverside *Odontesthes regia regia* and black cusk-eel *Genypterus maculatus* were associated with the ‘no’ category, suggesting lower bycatch risks.

## 4. Discussion

Based on rapid assessment surveys of nearly 800 fishers from 39 fishing ports in Peru and Chile, this study estimated Humboldt penguin bycatch by surface gillnet, bottom gillnet and purse-seine small-scale fisheries. In total 4067 (±889 s.d.) penguins were estimated captured between May 2022 and May 2023 (table 1). However, the mortality rate remains unknown, as many fishers declined to answer whether the penguins were caught dead or alive. While this bycatch estimation is high, penguin census data show that that level of catch is possible. In Peru, Altamirano *et al.* [33] counted a total of 15 663 penguins. In Chile, Wallace & Araya [34] estimated a penguin population of 33 284 but in later studies, this number has progressively decreased [8,10,35]. This study shows that gillnet and purse-seine fisheries pose sizeable, ongoing threats to Humboldt penguin populations in both countries.

### 4.1. Humboldt penguin threats

It is important to highlight that towards the end of 2022, Humboldt penguin populations were also impacted by avian flu (H5N1) (N°0180-2022-MIDAGRI-SENASA and N°0028-2023-MIDAGRI-SENASA for Peru; [36] for Chile) and an ENSO event [37], both of which have probably impacted many penguin colonies—demonstrated in observed population reductions in early 2023 (SENASA 2024, Servicio Nacional de Sanidad Agraria del Perú, unpublished data; AGRORURAL 2024, Programa de Desarrollo Productivo Agrario Rural, unpublished data; and [38]). Previous studies have also shown that ENSO events usually result in population declines at colonies owing to a higher dispersion to better foraging areas [7,10,39].

Overall, and in line with previous research, our results confirm that gillnet and purse seine small-scale fisheries have significant and concerning levels of penguin bycatch [16,19,40]. Our work also provides further evidence that, at least in Peru, part of the mortality related to fishery bycatch occurs because of the retention of penguins for human consumption (tables 3 and 4; [12,16]). Moreover, fisheries bycatch and mortality could continue to increase as human presence and fishing activity along the coast continue to expand [16]. It is important to highlight that the data presented here was obtained through surveys, a method recognized for its use in data-poor topics but also subject to various forms of potential bias (e.g. social desirability bias, under-reporting bias, knowledge bias, and others) [41,42]. For that reason, the bycatch reported could reflect either under- or over-estimations of the true bycatch rates. However, given the uncertainty, we cannot determine the direction of the bias, and we acknowledge this study as an important initial step towards future, more refined assessments of penguin bycatch.

### 4.2. Variation between fishing gear

Our results indicated that Humboldt penguin bycatch occurred in the three fishing gears we assessed, sometimes in sizeable numbers. In Peru, both surface and bottom gillnets had penguin bycatch during 2023, whereas the highest penguin bycatch in Chile was found in surface gillnets (table 1).

The interaction and impact of gillnets has been described in Peru, with a study near the Punta San Juan colony which showed that from total surface gillnets trips, 76% had bycatch (including mammals, turtles and seabirds, including Humboldt penguins), while 17% bottom gillnets trips reported bycatch [4]. Furthermore, the reports that penguins are often kept to be eaten or sold in Peru have also been described previously [16]. In Chile, the gillnet fishery has also been identified as a serious threat for penguin populations with the highest impact related to surface gillnets and bottom gillnets to a lesser degree [12,15].

Little information about the impact of bycatch in the purse-seine fishery on the penguin populations is available. In general, they have lower reported bycatch rates of seabirds compared to gillnets, a result that coincides with findings from our previous studies [19,43]. Several studies in central Chile indicated that seabird mortality in purse seines impacted primarily Magellanic penguins (*Spheniscus magellanicus*) and sooty shearwaters (*Ardenna grisea*) while Humboldt penguins were affected to a lesser degree [44,45]. Those studies highlighted that the factors relevant to seabird bycatch with purse seines include latitude, fleet size, and month of activity. Non-response rates for penguin condition and fate were notably high (tables 3 and 4) in our study, suggesting a possible reluctance by fishers to share this sensitive information. Despite this, the responses that were obtained present coherent trends that support the existence of penguin bycatch as a lethal threat, particularly by gillnets.

The interaction of penguins with gillnets and purse-seines ending in entanglements are suspected to occur when penguins are actively foraging, when nets are set in their path of travel, or when they are resting at the ocean surface [4,12,15,46]. It is common for seabirds to interact with fishing vessels owing to different factors such as the bait used or by targeting the same species [19,47]. Spheniscus penguins (e.g. Humboldt penguins) may have high entanglement probability in specific fisheries because of the overlap between the penguin diet (sardines and anchovies) and the fishery target species. [48]. In the Chilean purse-seine fishery targeting Peruvian anchovy *Engraulis ringens*, Araucanian herring *Strangomera bentincki* and Jack mackerel *Trachurus murphyi*, a high level of penguin bycatch has been reported [44,45]. However, our results showed a lower reported bycatch rate when the target species were Chilean silverside *Odontesthes regia regia* or Peruvian anchovy *E. ringens*, species related to a mesh size of 1–2 in (2.5 - 5.1 cm). Furthermore, when the target species were those that are not part of the Humboldt penguin diet [49] (e.g. Peruvian grunt *Anisotremus scapularis*, southern rock seabass *Paralabrax humeralis*, common dolphinfish *Coryphaena hippurus*), we found that the probability of a bycatch event occurring was high (see table 5 for the target species identified by fishers). Thus, our data were not able to clarify if penguin bycatch events were related to fisheries targeting prey of Humboldt penguins.

**Table 5.** Target species mentioned by surveyed fishers.

English name	Spanish name	scientific name
albacore	albacora	*Thunnus alalunga*
black cusk-eel	congrio	*Genypterus maculatus*
cabinza grunt	cabinza	*Isacia conceptionis*
Chilean silverside	pejerrey	*Odontesthes regia regia*
chub mackerel	caballa	*Scomber japonicus*
common dolphinfish	dorado - perico	*Coryphaena hippurus*
Corvina drum	Corvina	*Cilus gilberti*
Eastern Pacific bonito	Bonito	*Sarda chilensis*
Flathead grey mullet	Lisa	*Mugil cephalus*
Jack mackerel	Jurel del Pacífico sur	*Trachurus murphyi*
Jumbo squid	Jibia - Calamar gigante	*Dosidicus gigas*
Lorna drum	Lorna	*Sciaena deliciosa*
Pacific guitarfish	Guitarra	*Pseudobatos planiceps*
Palm ruff	Cojinova	*Seriolella violacea*
Peruvian anchovy	Anchoveta peruana	*Engraulis ringens*
Peruvian banded croaker	Suco	*Paralonchurus peruanus*
Peruvian flounder	Lenguado peruano	*Etropus peruvianus*
Peruvian grunt	Chita	*Anisotremus scapularis*
Peruvian hake	Merluza	*Merluccius peruanus*
Peruvian morwong	Pintadilla	*Chirodactylus variegatus*
Peruvian weakfish	Cachema	*Cynoscion analis*
Rays	Raya	Various spp.
Sharks	Tiburón	Various spp.
Snakehead kingcroaker	Bobo	*Menticirrhus ophicephalus*
South Pacific hake	Merluza	*Merluccius gayi*
Southern rock seabass	Cabrilla	*Paralabrax humeralis*

Although the reasons behind the interactions between Humboldt penguins and gillnet fisheries are variable [47], fishing net mesh size seems to be a more important factor related to an entanglement event than target species. Our results showed that nets with small mesh sizes (*ca* 1 in (2.5 cm)) had lower reported bycatch, but nets with mesh sizes between 3 and 6 in (7.6 cm and 15.2 cm) had higher reported bycatch. A tendency related to mesh size and target species has also been reported previously. Majluf *et al.* [4] showed that the palm ruff fishery with a mesh size of *ca* 5 in (12.7 cm) had the highest penguin bycatch in Punta San Juan. Simeone *et al.* [15] and Skewgar *et al.* [50] noted that the gillnet fisheries with corvina drum and South Pacific hake as target species, with a greater than 2 in. (5.1 cm) mesh size, are a direct and serious threat to penguin populations. Although our results show high values in estimated bycatch related to a greater than 2 in (5.1 cm) mesh size, it should also be taken into account that in San Jose and Pacasmayo (Peru) and Antofagasta (Chile), the majority of fishers reported mesh sizes larger than 2 in (5.1 cm). Additionally, while there is a relationship between ‘yes’ and 9 in (22.9 cm) or greater mesh size (ms9) apparent in figure 2, we consider this to be an ambiguous finding derived from very few data points, making interpretation difficult.

### 4.3. Variation between areas

Peruvian census information from Altamirano *et al.* [33] and AGRORURAL (2024, unpublished data) show that penguin populations are variable over months and between geographical areas. However, there are some areas that consistently have the highest values of penguin populations in almost every month. In Peru, these areas include Macabi island, North Guañape island, Melchorita (Peru-Liquid Natural Gas pier), Santa Rosa Island, Punta San Juan, Macabi and North Guañape islands. These areas are close to various ports evaluated in this study, specifically Pacasmayo, Salaverry and Chimbote, which all had an estimated mean of approximately one penguin bycatch per vessel per year. The areas around Macabi and North Guañape island were also described by fishers as areas with a high probability of penguin bycatch, highlighting the importance of the area (figure 1A). On the other hand, Melchorita and its surroundings were not described as an area with penguin bycatch (figure 1B). Melchorita is a private natural gas facility, undisturbed and protected, thus having better conditions on land and at sea for the establishment and growth of its Humboldt penguin population [38]. However, close to the pier are the ports Tambo de Mora, Pucusana and Ancon, which also have high reports of penguin bycatch. Therefore, even though fishers cannot approach the Melchorita pier, interactions with penguins from that colony could occur during foraging excursions since Humboldt penguins are capable of moving from 3 to 77 km d^−1^ to more than 140 km [8,46,51]. Finally, Punta San Juan is an important colony for penguins in Peru. This guano cape is near the port San Juan de Marcona which had a low level of estimated penguin bycatch compared to other ports in Peru. However, many fishers described a wide geographical area from Punta San Juan to Punta La Chira where penguin bycatch events occur (figure 1B). This may be a foraging area where penguins overlap with fishing vessels, which typically target palm ruff [4].

The north of Chile has been described as the area with the lowest penguin population in the country, specifically between 18° S and 26° S [10]. The largest population of Humboldt penguins in Chile is found along the north central coast (29° S–31° S) with Pajaros 1 island described as the most important colony [7]. Our results show Coquimbo and Tongoy, ports to the south of Pajaros 1 island, with a high estimated mean penguin bycatch per vessel, with Coquimbo being the port with the highest scaled bycatch in the study (table 2; figure 1D). Moreover, fishers described the area around Coquimbo as an important area of penguin bycatch. Finally, our results also show a wide area between 33° S and 34° S, where the principal ports with penguin bycatch were San Antonio and San Pedro Los Vilos. This area is near the colonies Islote Pajaro Niño, Isla Cachagua and Isla Huevos. In a 2016 census, Isla Cachagua hosted 456 breeding pairs [10] which could be related to an area of higher frequency of bycatch. Also, there is evidence of penguin bycatch by the purse-seine fishery in this area, although reported levels are low [10,44,45]. Whether the presence and quantity of bycatch we report could be derived or supported by these colonies requires further investigation.

### 4.4. Conclusions and recommendations

While our study provides valuable insights into penguin bycatch, we acknowledge certain potential sources of data bias. The bycatch estimates were scaled according to the fleet sizes reported for the ports, so inaccuracies in the numbers of active fishing vessels in these fleets will affect the bycatch estimates. Additionally, as has been suggested by other studies [52,53], the low fisher response rates to some questions generated potential under-reporting and shows that fishers are aware of the potential risks of reporting sensitive information like bycatch events. Some fishers may have chosen not to share information about bycatch to avoid legal trouble or social criticism, which would affect our results, such as by under-estimating rates of bycatch or mortality [41,42]. Despite these limitations, interview-based methods remain a critical tool in data-poor regions, offering a cost-effective approach towards identifying research and conservation priorities [24,41,42,54].

The interaction between penguins and fisheries also remains poorly understood. Our results indicate that the fishery target species may not be as clear an indicator of bycatch risk as previously thought. Rather, the mesh size of the nets, particularly those exceeding 3 in (7.6 cm), may play a crucial role in determining the rate of bycatch. Also, this assessment targeted small-scale fishing gears known to impact penguins, however industrial fisheries, in particular purse-seiners, might also have significant interaction risks with penguins that require additional research and monitoring [55].

This work, based on surveys, provides a baseline assessment for penguin bycatch in the two countries where most of its population occurs. However, to enhance the understanding of penguin bycatch, further focused research is essential, including using independent onboard observers, mark-recapture methods, and in particular, to identify solutions or otherwise mitigate the risk of injury and mortality resulting from bycatch. Political action is also necessary. In Chile, there is a National Plan of Action (NPOA, [56]) for reducing seabird bycatch which focuses on management actions to reduce bycatch rates. However, this NPOA was applied only to the longline fishery. Peru, on the other hand, is in the process of preparing a Plan de Conservacion for Humboldt penguins coordinated by the Servicio Nacional Forestal y de Fauna Silvestre (SERFOR). A seabird NPOA has yet to be addressed by the Peru government. Since the species occurs in both countries, a coordinated bi-national action plan or coordinated research activities for the species could also facilitate a comprehensive approach to protect penguin populations throughout their range, ensuring that management measures are applied that effectively mitigate the risk of bycatch.

## Data Availability

Our data are available for review at the following link [57]. Supplementary material is available online [58].
